# Tuberculosis Treatment Outcomes and Its Predictors among Tuberculosis Patients Registered at Tefera Hailu Memorial General Hospital, Sekota Town, Northeast Ethiopia: A Seven-Year Retrospective Study

**DOI:** 10.1155/2023/4212312

**Published:** 2023-03-06

**Authors:** Habtu Debash, Jemberu Nega, Habtye Bisetegn, Gebru Tesfaw, Daniel Getacher Feleke, Hussen Ebrahim, Alemu Gedefie, Mihret Tilahun, Ousman Mohammed, Ermiyas Alemayehu, Melaku Ashagrie Belete, Abdurahaman Seid, Agumas Shibabaw

**Affiliations:** ^1^Department of Medical Laboratory Science, College of Medicine and Health Sciences, Wollo University, Dessie, Ethiopia; ^2^Department of Medical Laboratory Science, Tefera Hailu Memorial General Hospital, Sekota, Ethiopia; ^3^Department of Internal Medicine, School of Medicine, Wollo University, Dessie, Ethiopia; ^4^Department of Microbiology, Immunology and Parasitology, College of Health Sciences, Addis Ababa University, Addis Ababa, Ethiopia

## Abstract

**Background:**

Despite the availability of effective medications, tuberculosis (TB) continues to be a serious global public health problem, primarily affecting low and middle-income nations. Measuring and reporting TB treatment outcomes and identifying associated factors are fundamental parts of TB treatment. The goal of this study was to look at the outcomes of TB treatment and the factors that influence them in Sekota, Northeast Ethiopia.

**Materials and Methods:**

A facility-based retrospective study was conducted in Tefera Hailu Memorial General Hospital, Sekota town, Northeast Ethiopia. All TB patients who registered in the TB log book and had known treatment outcomes at the treatment center between January 1, 2015, and December 30, 2021, were included in this study. The data was gathered utilizing a pretested structured data extraction format that comprised demographic, clinical, and treatment outcome characteristics. Data were entered, cleaned, and analyzed using SPSS version 25. Descriptive statistics and logistic regression analysis were employed. A *p* value of less than 0.05 was considered statistically significant.

**Results:**

A total of 552 registered TB patients' data were reviewed. Of these, 49.6% were male, 94.4% were new cases, 64.9% were presented with pulmonary TB, and 18.3% were HIV positive. Regarding the treatment outcome, 11.6% were cured, 82.2% completed their treatment, 1.1% had failed treatment, 1.3% were lost to follow-up, and the remaining 3.8% died during the follow-up. The overall treatment success rate among TB patients was 93.8%. The maximum number of successful treatment outcomes was 94.9% in 2021, while the lowest was 86.7% in 2020. The pattern of successful treatment results changes with the number of years of treatment. In the current study, being a new TB patient (AOR = 1.75, 95% CI: 1.31–7.32) and being an HIV-negative patient (AOR = 2.64, 95% CI: 1.20–5.8) were factors independently associated with a successful treatment outcome.

**Conclusion:**

The rate of successful TB treatment outcomes in the current study was satisfactory. This achievement should be maintained and enhanced further by developing effective monitoring systems and educating patients about medication adherence.

## 1. Introduction

Tuberculosis (TB) continues to be a serious public health issue worldwide, accounting for the 13th leading cause of death and the second leading infectious killer after COVID-19 [1]. Although highly effective treatment has been available for decades, TB remains a challenge, especially in low-income countries [2]. Tuberculosis epidemiology is also closely connected with social and economic conditions, which makes its prevention, care, and control more challenging [3]. In 2021, a projected 10.6 million people became ill with tuberculosis, up from 10.1 million in 2020, and 1.6 million people died from tuberculosis, including 187,000 people living with HIV [1]. Tuberculosis is the leading cause of death among communicable diseases in Ethiopia [4].

In Ethiopia, early detection, diagnosis, and treatment of TB cases have been practiced for years as per the guidelines of the Directly Observed Treatment Short-course (DOTS) program that started in 1997 [5]. Despite the DOTS program's adoption, many reports from around the country have suggested that there are obstacles to improving TB treatment outcomes. These include differences in treatment-seeking behavior, poor compliance, the occurrence of HIV/TB coinfection, discrepancies in expert qualifications, and the presence of medication resistance [6, 7].

According to the Ethiopian national TB and leprosy control strategic plan, it has been planned to successfully treat 90% of confirmed TB cases and enroll them in the DOTS program. Moreover, regarding the 2020 Global TB Report, the successful TB treatment outcome in Ethiopia was 88% by the year 2019 [8]. Such results have only been obtained with vigorous treatment and follow-up. Several studies [9–11] conducted in various parts of Ethiopia revealed varying rates of treatment effectiveness and associated factors.

Determining TB trends and treatment outcomes in health facilities is critical for better disease management and control efforts. Nonetheless, data from Ethiopia's rural, urban, and suburban environments demonstrate heterogeneity and inconsistency. Routine monitoring of the extent of the treatment outcome and its drivers is vital; however, studies on TB treatment outcomes and associated factors are rare in Northeast Ethiopia, particularly in the Waghemra zone. As a result, the findings of this study are critical for the study area as well as at the national level in order to reduce the burden and identify predictors of good treatment outcomes. As a result, the purpose of this study was to evaluate TB treatment outcomes and associated factors at Tefera Hailu Memorial General Hospital in Sekota town.

## 2. Materials and Methods

### 2.1. Study Design, Area, and Period

A facility-based retrospective study was conducted in Tefera Hailu Memorial General Hospital in Sekota town, the capital of Waghemra zone, Amhara Regional State, Ethiopia. Tefera Hailu Memorial General Hospital is found in the Northeast part of Ethiopia, 720 km from the capital city of Addis Ababa. It serves a total population of 536,129. The hospital provides all types of clinical and diagnostic services, as well as supervisory and mentoring services, to the nearby health centers found in the Waghemra zone. It is the center for TB diagnosis and treatment, including multi-drug or extended drug resistance TB (MDR/XDR-TB) patient treatment, care, and management. TB patient registration documents were reviewed from January 1, 2015, to December 30, 2021. The study period to review the seven-year (2015–2021) data was from January 1 to April 30, 2022.

### 2.2. Study Population

All TB patients who started treatment in the DOTS clinic and had complete medical records on TB treatment outcomes at Tefera Hailu Memorial General Hospital in the past seven years were included in the study. Patients who had incomplete data from the logbook were excluded from the study.

### 2.3. Sample Size and Sampling Technique

A total of 589 tuberculosis patients were treated from 2015 to 2021 at Tefera Hailu Memorial General Hospital. Then, 552 patients who fulfilled the inclusion criteria were included in the study.

### 2.4. Data Collection

#### 2.4.1. Data Collection Tool

A structured data abstraction checklist was created and used to extract pertinent information. For content validity, the checklist was pretested and standardized among 5% of the study population at Dehana District Hospital. A data abstraction checklist based on both dependent and independent factors was created. The checklist covers sociodemographics, HIV status, ART status, CPT initiation, tuberculosis type, patient category, and therapy outcome.

#### 2.4.2. Data Collection Procedure

Data were gathered from a secondary source, the TB patients' registration record book. In the *tuberculosis* clinic, statistics were gathered through medical record reviews of patients using a pre-prepared standard checklist. Supervisors for data collection included two BSc nurses and one laboratory technologist. The TB registry was used to obtain demographic and clinical information such as age, gender, type of *tuberculosis*, HIV status, cotrimoxazole preventive therapy (CPT), and ART status.

#### 2.4.3. Data Quality Control

The data collectors were instructed for one day about the goal and data abstraction techniques prior to the real data collection. Prior to the actual data collection at Dehana District Hospital, a 5% pretest was conducted to evaluate the validity of the data abstraction checklist. As a result, changes and adjustments were made to the data abstraction checklist. The lead investigator also kept a close eye on the activity on a regular basis. Furthermore, prior to the start of the research, the lead investigator meticulously entered and cleaned the data.

### 2.5. Definitions of Outcome Terms

The clinical definition and treatment outcome of patients were recorded in accordance with the Ethiopian National TB and Leprosy Control Program (NTLCP) standards and WHO recommendations [12]. In this study, therapy outcomes are classified as successful or unsuccessful. Cases with a successful treatment outcome included “cured” and “treatment completed” cases. In contrast, unsuccessful treatment outcomes included “treatment failure” cases, “defaulters,” and patients who “died.” The patient is deemed cured if he or she completes treatment with a negative bacteriological result at the end of the treatment. The treatment is considered “complete” if the patient completed treatment but did not receive a bacteriological result. Treatment failure: a patient who remained smear-positive during treatment or relapsed five months later; or a patient who was PTB negative at the start but became smear-positive at the end of the intensive phase. A defaulter is a patient who has been on treatment for at least four weeks and has had two or more weeks of therapy interrupted in a row. The patient died as a result of any cause during the course of treatment.

### 2.6. Statistical Analysis

After checking the data for completeness and consistency, the data were entered and analyzed using SPSS version 25. The socio-demographic and clinical characteristics of the study participants are presented using descriptive statistics. The findings are presented using frequency tables, graphs, and percentages. Bivariate and multivariate logistic regression analyses were used to determine the relationship between dependent and independent variables. Explanatory variables with a *p* value of <0.25 in the bivariate analysis were included in the multivariate logistic regression model. A *p* value of <0.05 was considered statistically significant. The assumption of model fitness was checked by the Hosmer and Lemeshow test (*p*=0.752).

## 3. Results

### 3.1. Socio-Demographic Characteristics of TB Patients

All TB patients who started treatment in the DOTS clinic and documented their registration in the TB log book at Tefera Hailu Memorial General Hospital from January 1, 2015, to December 30, 2021, were included in this study. A total of 589 TB patients were treated from 2015 to 2021 at Tefera Hailu Memorial General Hospital. Of these, 552 had known treatment outcomes that were included in the study. However, 37 TB patients with incomplete data were excluded from the study. From the total participants included in this study, 278/552 (50.4%) cases were female, 175/552 (31.7%) were between 30 and 44 years old, 331/552 (60.0%) were urban dwellers, and 272/552 (49.3%) weighed 38 to 54 kg (Table 1).

### 3.2. Clinical Characteristics of TB Patients

Regarding the TB category, 521/552 (94.4%) were new TB cases. Three hundred fifty-eight (358/552 (64.9%)) of the patients presented with pulmonary TB, and of these, 70/358 (19.6%) were smear-positive. A significant number of the patients, 101/552 (18.3%), were HIV-TB coinfected patients; of these, 100/101 (99.0%) and 99/101 (98.0%) were on ART and took cotrimoxazole preventive treatment (CPT), respectively. During the intensive phase of treatment, 548/552 (99.3%) of TB patients received a combination of rifampicin, isoniazid, pyrazinamide, and ethambutol (RHZE) as part of their treatment regimen. Moreover, all patients were treated with the standard RH regimen during the continuation phase of therapy (Table 2).

### 3.3. Trends in Types of TB Cases across the Seven Years

**Figure 1:**
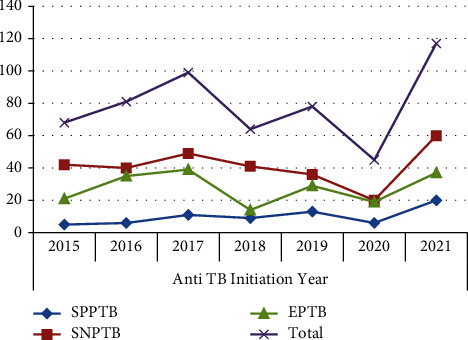
Trends of TB types diagnosed at Tefera Hailu Memorial General Hospital, Sekota town, Northeast Ethiopia from 2015 to 2021.

The trend for all forms of TB showed a fluctuating trend. From 2015 to 2017, the number of smear-positive PTB, smear-negative PTB, and EPTB cases increased. However, all forms of TB showed a slight decline from 2017 to 2020 with a certain fluctuation, whereas the number of TB cases increased in the last year of 2021 (Figure 1).

### 3.4. Treatment Success and Associated Factors

The overall rate of successful TB treatment outcomes (cured and treatment completed) among patients enrolled in the DOTS program was 518/552 (93.8%). Of these, 64/552 (11.6%) were cured, and 454/552 (82.2%) had completed their treatment. The treatment success rate of HIV-coinfected TB patients included in the TB treatment outcome was 87/101 (86.1%). From a total of 34/552 (6.2%) unsuccessful TB treatment outcome cases, 6/552 (1.1%) had failed treatment, 7/552 (1.3%) were lost to follow-up, and the remaining 21/552 (3.8%) died during the follow-up. Patients from rural areas, women, EPTB patients, and HIV-negative patients had higher treatment success rates. The cure rate showed an increment during the review period, from 5/68 (7.4%) in the year 2015 to 18/117 (15.4%) in the year 2021. The death rate also showed an increment for six consecutive years (2015–2020) and a decline in 2021. However, the rate of treatment completion showed a reduction in the six years (2015–2020) from 61/68 (89.7%) to 32/45 (71.1%) and then started to increase in the final year of the study period (2021) (Table 3).

**Figure 2:**
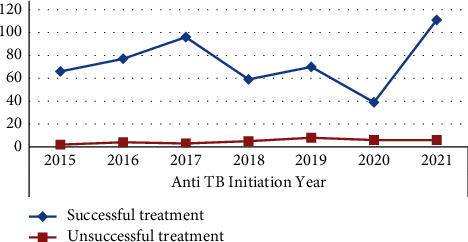
Trend of successful TB treatment outcomes of TB patients at Tefera Hailu Memorial General Hospital, Sekota, Northeast Ethiopia from 2015 to 2021.

The maximum number of successful treatment outcomes was 111/117 (94.9%) in 2021, while the lowest was 39/45 (86.7%) in 2020. The pattern of successful treatment results changes with the number of years of treatment. This means that the trend of successful TB treatment results rose gradually from 2015 to 2017. However, the number of TB patients who had successful treatment varied greatly from 2017 to 2020, peaking in 2021 (Figure 2).

In the bivariate analysis, variables with a *p* value of <0.25 were age, pretreatment weight, year of treatment initiated, type of TB, patient category, and HIV status. In the multivariate logistic regression, TB patient category and HIV status were independently and significantly associated with the treatment outcome. The odds of treatment success were 1.75 (AOR = 1.75, 95% CI: 1.31–7.32) times higher among new TB patients than retreatment TB cases. Similarly, HIV-negative patients had 2.64 times higher odds of (AOR = 2.64, 95% CI: 1.20–5.8) having treatment success than HIV-positive patients (Table 4).

## 4. Discussion

An institution-based seven-year retrospective study was done to extract and review 552 registered TB patients at the DOTS clinic. Females were nearly equal to males in this study, accounting for 50.4%. In contrast to this study, previous studies reported that males were dominant, such as a study in Dessie [9], Goba [10], and Harar [11]. The burden of TB was higher among the age groups of 15–29 and 30–44 years, with 28.4% and 31.7%, respectively, which was in agreement with the previous study in southern Ethiopia [13]. This might be indicating a negative influence of TB on the socioeconomic state of society, particularly in low-income countries.

Of all patients enrolled in the DOTS clinic, 18.3% were HIV-positive, comparable with the reports done in Woldia (18.5%) [14], East Gojam (22.9%) [6], Goba (17.4%) [10], and Harar (22.8%) [11]. Human immunodeficiency virus-positive individuals with low immunity might be coinfected with a variety of pathogens, including TB, in varying degrees of clinical manifestations. Previous studies in Debre Tabor (12.7%) [15], Gondar (13.4%) [16], Tigray region (8.6%) [17], Iran (2.7%) [18] and Malaysia (6.6%) [19] found lower HIV-TB coinfection. This high TB-HIV coinfection in our study could be due to the higher proportion of HIV in urban settings, where the majority of the study participants lived. More than half of the participants (52.2%) had smear-negative pulmonary TB, which was higher than the study reported in East Gojam (42%) [6], and Wolyta Sodo (34.5%) [13]. Patients who came to the health facility with TB signs and symptoms and had a negative result for AFB microscopy were clinically diagnosed as smear-negative PTB. This smear microscopy-based detection might contribute to lowering the rate of smear-positive PTB cases, as most of the health facilities in Ethiopia have been using AFB microscopy due to limited resources. Smear microscopy has low sensitivity (40–45%) and might contribute to lower notification rates of smear-positive PTB [20].

The trend analysis result showed that TB cases were fluctuating over the past seven years but ultimately showed an increment in the most recent year. Other studies reported in Northern Ethiopia [21] and the Sidama Zone [22] found a similar fluctuating but increasing trend. Different factors may contribute to the variation of TB cases, including altitude variations, ecological and natural variables, social and economic factors, control efforts, population vulnerability, and drug resistance [23].

Although trends in the number of cases vary by year, 2020 showed a significantly lower number of cases. This could be attributed to the reorganization of hospitals and health centers for COVID-19 care, detection, and isolation; patients' fear of being isolated if their infection is COVID-19; patients' fear of contracting COVID-19 in health facilities; and the reallocation of healthcare professionals for COVID-19 care and management in Ethiopia during the pandemic in 2020. An increase in the number of cases was observed since 2017 and reached its peak in 2021. A considerable decline in TB case detection due to missed diagnoses has resulted in an accumulation of undiscovered TB, resulting in continued TB transmission and higher rates of latent TB infection. Moreover, huge numbers of people immigrated from their local areas to Sekota town due to internal conflict in Ethiopia. This also might be due to the high rates of TB transmission among homeless persons, injection drug users, and persons with HIV infection, according to the WHO protocol [24].

The overall magnitude of successful TB treatment outcomes was 93.8% (95% CI, 91.8–95.7). Of these, 11.6% were cured, and 82.2% completed their treatment. The overall successful treatment outcome recorded in the present study was in agreement with the retrospective studies conducted in Gojam, Northwest Ethiopia, 94.8% [25], 92.4% in Hawassa [26], 92.5% in Harar [11], 91.9% in East Wollega [27], 94.9% in Pakistan [28], 91.7% in Iran [29], 95.1% in Russia [30], and the WHO 2030 international target of 90% [8].

On the contrary, the successful treatment outcome of this study was higher than reports from other studies, with a treatment success rate of 80.7% in Woldia [14], 90.1% in Debre Tabor [20], 89.5% in Northern Ethiopia [21], 91.2% in Goba (91.2%) [10], 80.4% in Tepi [31], 82.5% in Wolayta Sodo [13], 86.0% pooled prevalence in Ethiopia [7], 75.7% in Sudan [32], 57.7% in Nigeria [33], and 50.7% in China [34]. This could be due to improved patient adherence to anti-TB medication, an increased focus on TB prevention and control programs in our study, and changes in study duration.

In our findings, 6.2% of TB patients had an unsuccessful or poor TB treatment outcome. Poor adherence to anti-TB treatment due to treatment failures lost to follow-up and irregular treatment might lead to more severe illness, treatment failure, relapse, a longer infection, drug resistance, and even death. Defaulting and irregular anti-TB medicine intake presented a problem and a worry for both the patient and the community and needed to be addressed appropriately. One of the most difficult issues during the TB control campaign is finding treatment failures and ensuring follow-up. The rate of treatment failure among TB patients was 1.1%, which was lower than the findings documented in Debre Tabor (3.5%) [15] and in Tigray Region (3.7%) [17]. Our study's lower treatment failure rate could be attributed to better implementation of DOT strategies such as defaulter tracing, supervision, and health education activities for inpatients, outpatients, and the community.

In our study, 1.3% of the TB patients were identified as lost to follow-up. The findings were similar to those of similar studies conducted in Hosanna (1.4%) [35] and Sidama, Ethiopia (1.0%) [22]. This figure was lower than the reports in Northern Ethiopia (2.5%) [36], Hawassa (2.6%) [26], Wolayta Sodo (11.2%) [13], Harar (2.4%) [11], Dilla (11.1%) [37], and East Wollega (2.9%) [27]. This low loss to follow-up rate could be attributed to the community's effective execution of the DOT's plan, counseling services, and good healthcare utilization and seeking behavior. In addition, the TB-related death rate was 3.8% in this study. This figure was in line with reports in Gondar (3.27%) [38], Tigray Region (3.9%) [17], Addis Ababa (3.7%) [39], and Harar (3.9%) [11]. However, this finding was lower than the mortality rates reported for Wolayta Sodo (4.7%) [13], Goba (5.1%) [10], Nigeria (11.5%) [40], and Yemen (12.2%) [41]. This could be explained by greater access to and utilization of TB control services, as well as increased disease awareness in our study area.

According to our findings, patients treated in 2021 have a higher chance of a successful TB treatment outcome than those treated between 2015 and 2020. This finding is supported by a study conducted at Woldia [14], Awi Zone, Northwest Ethiopia [42], and Addis Ababa [43]. This could be due to the patient's better awareness of the treatment schedule, improved adherence to anti-TB treatment, and receiving better care over time. Identification of factors related to TB treatment results is critical in order to address the factors responsible for poor treatment outcomes. Our findings revealed that being a new TB case and HIV-negative was independently and significantly associated with successful treatment outcomes.

New TB cases were nearly twice as likely as their counterparts to have successful treatment outcomes. This finding was in agreement with studies conducted in the Arsi Zone, Ethiopia [44], Somalia [45], Nigeria [40], and Turkey [46]. Recurrence instances, on the other hand, could be drug-resistant TB, a recurrence due to a weakened immune system, or other comorbidities that enhance the probability of an unsatisfactory treatment outcome. Furthermore, individuals who had HIV-negative TB patients were nearly three times more likely than HIV-positive patients to have good treatment outcomes. The finding was similar to the study in Woldia [14] and in Harar, Eastern Ethiopia [11]. This could be explained by the fact that HIV-negative TB patients have better immune systems than HIV-positive patients, and HIV-positive patients may not take the medicine as prescribed due to concerns about drug interactions and side effects [47].

### 4.1. Limitation of the Study

As retrospective secondary data was obtained from the TB registration log book, it was not possible to determine all factors that could affect the patient's treatment outcomes. Moreover, since the study was conducted in a single hospital, it was difficult to generalize the findings to all TB patients in the country.

## 5. Conclusion and Recommendation

The success rate of TB treatment in the study area was generally good when compared to other studies conducted in Ethiopia, and it was higher than the aim established by the national TB and leprosy control program in Ethiopia. Being a new TB case and being HIV-negative were both predictors of effective treatment outcomes. Early detection of TB and the timely beginning of effective anti-TB drug treatment, as well as increased HIV prevention and health education activities, are critical for improving the treatment outcomes of TB patients. Furthermore, more prospective studies are needed to determine other predictors that may affect TB patient' treatment success.

## Figures and Tables

**Table 1:** Sociodemographic characteristics of TB patients at Tefera Hailu Memorial General Hospital, Sekota town, Northeast Ethiopia from January 2015 to December 2021.

Characteristic		Frequency (*n*)	Proportion (%)
Sex	Male	274	49.6
	Female	278	50.4

Age category	<15	55	10.0
	15–29	157	28.4
	30–44	175	31.7
	45–59	110	19.9
	≥60	55	10.0

Pretreatment weight	Less than 20 kg	29	5.3
	20–29 kg	36	6.5
	30–37 kg	121	21.9
	38–54 kg	272	49.3
	>54 kg	94	17.0

District	Sekota town	391	70.8
	Sekota zuria	148	26.8
	Others	13	2.4

Residence	Urban	331	60.0
	Rural	221	40.0

Year of treatment	2015	67	12.1
	2016	82	14.9
	2017	99	17.9
	2018	64	11.6
	2019	78	14.1
	2020	45	8.2
	2021	117	21.2

**Table 2:** Clinical characteristics of TB patients at Tefera Hailu Memorial General Hospital, Sekota town, Northeast Ethiopia from 2015 to 2021.

Variables	Category	Frequency (*n*)	Proportion (%)
Type of TB	SPPTB	70	12.7
	SNPTB	288	52.2
	EPTB	194	35.1

TB category	New case	521	94.4
	Retreatment	31	5.6

HIV status	HIV-positive	101	18.3
	HIV-negative	451	81.7

Cotrimoxazole preventive therapy initiated	Yes	99	98.0
	No	2	2.0

ART initiated	Yes	100	99.0
	No	1	1.0

Treatment regimen during the intensive phase	RHZE	548	99.3
	SERHZ	4	0.7

**Table 3:** Distribution of treatment outcome with socio-demographic and clinical characteristics of TB patients at Tefera Hailu Memorial General Hospital, Sekota town, Northeast Ethiopia from 2015 to 2021.

Characteristics		Treatment outcome					Total
		Cured (%)	Treatment completed (%)	Treatment failed (%)	Lost to follow-up (%)	Died (%)	
Sex	Male	29 (10.6)	226 (82.5)	2 (0.7)	7 (2.6)	10 (3.6)	274
	Female	35 (12.6)	228 (83.2)	4 (1.4)	0 (0.0)	11 (4.0)	278

Age category	<15	2 (3.6)	49 (89.1)	0 (0.0)	2 (3.6)	2 (3.6)	55
	15–29	35 (22.3)	116 (73.9)	3 (1.9)	1 (0.6)	2 (1.3)	157
	30–44	19 (10.9)	140 (80.0)	1 (0.6)	3 (1.7)	12 (6.9)	175
	45–59	6 (5.5)	97 (88.2)	2 (1.8)	1 (0.9)	4 (3.6)	110
	≥60	2 (3.6)	52 (94.5)	0 (0.0)	0 (0.0)	1 (1.8)	55

Pretreatment weight	<20 kg	2 (6.9)	23 (79.3)	0 (0.0)	1 (3.4)	3 (10.3)	29
	20–29 kg	5 (13.9)	30 (83.3)	0 (0.0)	1 (2.8)	0 (0.0)	36
	30–37 kg	16 (13.2)	94 (77.7)	2 (1.7))	1 (0.8)	8 (6.6)	121
	38–54 kg	29 (10.7)	228 (83.8)	4 (1.5)	3 (1.1)	8 (2.9)	272
	>54 kg	12 (12.8)	79 (84.0)	0 (0.0)	1 (1.1)	2 (2.1)	94

District	Sekota town	56 (14.3)	310 (79.3)	6 (1.5)	4 (1.0)	15 (3.8)	391
	Sekota zuria	8 (5.4)	131 (88.5)	0 (0.0)	3 (2.0)	6 (4.1)	148
	Others	0 (0.0)	13 (100.0)	0 (0.0)	0 (0.0)	0 (0.0)	13

Place of residence	Urban	46 (13.9)	263 (79.5)	5 (1.5)	4 (1.2)	13 (3.9)	331
	Rural	18 (8.1)	191 (86.4)	1 (0.5)	3 (1.4)	8 (3.6)	221

Year of treatment	2015	5 (7.4)	61 (89.7)	1 (1.5)	1 (1.5)	0 (0.0)	68
	2016	5 (6.2)	72 (88.9)	1 (1.2)	2 (2.5)	1 (1.2)	81
	2017	12 (12.1)	84 (84.8)	0 (0.0)	1 (1.0)	2 (2.0)	99
	2018	8 (12.5)	51 (79.7)	1 (1.6)	0 (0.0)	4 (6.25)	64
	2019	9 (11.5)	61 (78.2)	2 (2.6)	1 (1.3)	5 (6.4)	78
	2020	7 (15.6)	32 (71.1)	0 (0.0)	0 (0.0)	6 (13.3)	45
	2021	18 (15.4)	93 (79.5)	1 (0.9)	2 (1.7)	3 (2.7)	117

Type of TB	SPPTB	61 (87.1)	2 (2.9)	3 (4.3)	0 (0.0)	4 (5.7)	70
	SNPTB	1 (0.3)	268 (93.1)	2 (0.69)	5 (1.7)	12 (4.2)	288
	EPTB	2 (1.0)	184 (94.8)	1 (0.5)	2 (1.0)	5 (2.6)	194

TB category	New case	57 (10.9)	432 (82.9)	6 (1.2)	6 (1.2)	20 (3.8)	521
	Retreatment	7 (22.6)	22 (71.0)	0 (0.0)	1 (3.2)	1 (3.2)	31

HIV status	HIV-positive	15 (14.9)	72 (71.3)	1 (1.0)	2 (2.0)	11 (10.9)	101
	HIV-negative	49 (10.9)	382 (84.7)	5 (1.1)	5 (1.1)	10 (2.2)	451

Total		64 (11.6)	454 (82.2)	6 (1.1)	7 (1.3)	21 (3.8)	552

**Table 4:** Bivariate and multivariate analysis of treatment outcome with socio-demographic and clinical characteristics of TB patients at Tefera Hailu Memorial General Hospital, Sekota town, Northeast Ethiopia from 2015 to 2021.

Variables		Successful TB treatment outcome					
		Yes	No	COR (95% CI)	*p* value	AOR (95% CI)	*p* value
		*N* (%)	*N* (%)				
Sex	Male	255 (93.1)	19 (6.9)	1			
	Female	263 (94.6)	15 (5.4)	1.31 (0.65–2.63)	0.453		

Age category	<15	51 (92.7)	4 (7.3)	1		1	
	15–29	151 (96.2)	6 (3.8)	4.24 (0.49–39.17)^*∗*^	0.203	1.32 (0.09–20.58)	0.842
	30–44	159 (90.9)	16 (9.1)	2.15 (0.25–18.23)^*∗*^	0.184	1.43 (0.16–12.88)	0.751
	45–59	103 (93.6)	7 (6.4)	5.43 (0.70–41.95)^*∗*^	0.105	3.48 (0.43–28.17)	0.242
	≥60	54 (98.2)	1 (1.8)	3.67 (0.44–30.61)^*∗*^	0.230	1.69 (0.46–6.16)	0.428

Pretreatment weight	<20 kg	25 (86.2)	4 (13.8)	1		1	
	20–29 kg	35 (97.2)	1 (2.8)	4.85 (1.02–23.12)^*∗*^	0.047	8.56 (0.94–77.86)	0.057
	30–37 kg	110 (90.9)	11 (9.1)	0.87 (0.09–8.61)^*∗*^	0.203	1.19 (0.09–15.17)	0.893
	38–54 kg	257 (94.5)	15 (5.5)	3.03 (0.82–11.20)^*∗*^	0.096	2.67 (0.69–10.40)	0.157
	>54 kg	91 (96.8)	3 (3.2)	1.77 (0.50–6.26)^*∗*^	0.375	1.688 (0.46–6.16)	0.428

Residence	Urban	309 (93.4)	22 (6.6)	0.81 (0.39–1.67)	0.561		
	Rural	209 (94.6)	12 (5.4)	1			

Year of treatment	2015	66 (97.1)	2 (2.9)	1			
	2016	77 (95.1)	4 (4.9)	0.56 (0.11–2.86)^*∗*^	0.216	0.94 (0.17–5.10)	0.945
	2017	96 (97.0)	3 (3.0)	0.96 (0.26–3.52)^*∗*^	0.152	1.28 (0.33–5.04)	0.721
	2018	59 (92.2)	5 (7.8)	0.58 (0.14–2.37)^*∗*^	0.147	0.74 (0.174–3.15)	0.683
	2019	70 (89.7)	8 (10.3)	1.57 (0.46–5.35)^*∗*^	0.123	1.965 (0.53–7.30)	0.313
	2020	39 (86.7)	6 (13.3)	2.11 (0.70–6.35)^*∗*^	0.082	2.48 (0.77–7.96)	0.1282
	2021	111 (94.9)	6 (5.1)	2.85 (0.87–9.35)^*∗*^	0.085	3.54 (0.99–12.69)	0.053

Type of TB	SPPTB	63 (90.0)	7 (10.0)	0.64 (0.26–1.58)^*∗*^	0.229	2.18 (0.69–6.85)	0.183
	SNPTB	269 (93.4)	19 (6.6)	0.39 (0.14–1.11)^*∗*^	0.078	1.37 (0.56–3.38)	0.491
	EPTB	186 (95.9)	8 (4.1)	1			

TB category	New case	489 (93.9)	32 (6.1)	1.05 (0.54–5.12)^*∗*^	0.144	1.75 (1.31–7.32)^*∗*^	0.020
	Retreatment	29 (93.5)	2 (6.5)	1			

HIV status	HIV-positive	87 (86.1)	14 (13.9)	1			
	HIV-negative	431 (95.6)	20 (4.4)	3.47 (1.69–7.13)^*∗*^	0.001	2.64 (1.20–5.80)^*∗*^	0.016

^*∗*^Statistically significant at *p* < 0.05.

## Data Availability

All relevant data are included in the published article.
